# Supplementary material for: Entropy production and non-Markovian dynamical maps
Source: Sci Rep. 2017 Sep 29;7:12447. doi: 10.1038/s41598-017-12595-x (PMC5622134; doi:10.1038/s41598-017-12595-x)
Supplement: Supplementary file 1 — Entropy production and non-Markovian dynamical maps [file 41598_2017_12595_MOESM1_ESM.pdf]

Entropy production and non-Markovian dynamical maps

S. Marcantoni[1,2], S. Alipour[3], F. Benatti[1,2,*], R. Floreanini[2], and A. T. Rezakhani[4]

[1]Department of Physics, University of Trieste, I-34151 Trieste, Italy
[2]National Institute for Nuclear Physics (INFN), Trieste Section, I-34151 Trieste, Italy
[3]School of Nano Science, Institute for Research in Fundamental Sciences (IPM), Tehran 19538, Iran
[4]Department of Physics, Sharif University of Technology, Tehran 14588, Iran
[*]benatti@ts.infn.it

ABSTRACT

Here we present some further details related to Example I.

Supplementary Information: Sign of the entropy production in Example I

From Eq. (18) in the main text it seems that the sign of $\boldsymbol{\sigma}_\tau$ depends both on γ_τ and on the sign of the expression within the square brackets. In this section we prove that the latter is always positive. Let us rewrite the entropy production in a more convenient way as

$$\boldsymbol{\sigma}_\tau = \gamma_\tau \coth(\beta\omega/2)\, \mathrm{e}^{-2\Gamma_\tau}[A+B+C],$$

where

$$A = \frac{x_0^2+y_0^2}{4r_\tau}\log\left(\frac{1+r_\tau}{1-r_\tau}\right), \quad B = \frac{z_0+|z_\infty|}{2}\log\left(\frac{1+r_\infty}{1-r_\infty}\right), \quad C = \frac{(z_0+|z_\infty|)z_\tau}{2r_\tau}\log\left(\frac{1+r_\tau}{1-r_\tau}\right).$$

Note that A is always nonnegative whereas B and C can be either positive or negative. We show in the following that $A+B+C$ is nevertheless positive, distinguishing different situations.

1. If $z_0+|z_\infty| \leqslant 0$, then $z_\tau \leqslant -|z_\infty|$, because $z_\tau + |z_\infty| = \mathrm{e}^{-2\Gamma_\tau}(z_0+|z_\infty|)$ and

$$|r_\tau| \geqslant |z_\tau| \geqslant |z_\infty| = r_\infty.$$

   Hence $B+C \geqslant 0$, because

$$r_\tau \log\left(\frac{1+r_\infty}{1-r_\infty}\right) - |z_\tau|\log\left(\frac{1+r_\tau}{1-r_\tau}\right) \leqslant r_\tau \log\left(\frac{1+r_\infty}{1-r_\infty}\right) - r_\infty\log\left(\frac{1+r_\tau}{1-r_\tau}\right) \leqslant 0,$$

   where the last inequality holds because the function

$$f(x) = \frac{1}{x}\log\left(\frac{1+x}{1-x}\right),$$

   is monotonically increasing for $0<x<1$. This can be seen from the first derivative,

$$f'(x) = \frac{1}{x^2}\left[\frac{2x}{1-x^2} - \log\left(\frac{1+x}{1-x}\right)\right],$$

   which is is always positive because

$$\frac{2x}{1-x^2} - |\log(1+x)| - |\log(1-x)| \geqslant \frac{2x}{1-x^2} - \frac{x}{2}\frac{2+x}{1+x} - \frac{x}{2}\frac{2-x}{1-x} = \frac{x^3}{1-x^2} \geqslant 0,$$

   in which the following inequalities have been used[1]:

$$\frac{2x}{2+x} \leqslant |\log(1+x)| \leqslant \frac{x}{2}\frac{2+x}{1+x}, \qquad \frac{2x}{2-x} \leqslant |\log(1-x)| \leqslant \frac{x}{2}\frac{2-x}{1-x}.$$

2. In the case $z_0 + |z_\infty| \geqslant 0$, we need to distinguish different situations.

   - First, if $z_\tau \geqslant 0$, then B and C are both positive.
   - If instead $-|z_\infty| \leqslant z_\tau \leqslant 0$ and $r_\tau \leqslant r_\infty$, then $B + C$ is positive because

     $$\log\left(\frac{1+r_\infty}{1-r_\infty}\right) - \frac{|z_\tau|}{r_\tau}\log\left(\frac{1+r_\tau}{1-r_\tau}\right) \geqslant \log\left(\frac{1+r_\infty}{1-r_\infty}\right) - \log\left(\frac{1+r_\tau}{1-r_\tau}\right) \geqslant 0.$$

   - The last possibility is $-|z_\infty| \leqslant z_\tau \leqslant 0$ and $r_\tau \geqslant r_\infty$. In this case, B is positive and the following inequality holds:

     $$x_0^2 + y_0^2 \geqslant (|z_\infty| - z_\tau)(z_0 + |z_\infty|) \geqslant 0.$$

     As a consequence, $A + C \geqslant 0$,

     $$x_0^2 + y_0^2 - 2|z_\tau|(z_0 + |z_\infty|) \geqslant x_0^2 + y_0^2 - (|z_\infty| + |z_\tau|)(z_0 + |z_\infty|) \geqslant 0.$$

Summarizing, the expression in the square brackets $[A + B + C]$ is always nonnegative and the sign of the entropy production is only determined by γ_τ.

References

1. Topsøe, F. Some Bounds for the Logarithmic Function. in: Cho, Y.J., Kim, J.K. & Dragomir, S.S., *Inequality Theory and Applications.* Nova Science Publishers, New York, 2007.
